# Sorghum Flour and Sorghum Flour Enriched Bread: Characterizations, Challenges, and Potential Improvements

**DOI:** 10.3390/foods12234221

**Published:** 2023-11-22

**Authors:** Saeed Hamid Saeed Omer, Jing Hong, Xueling Zheng, Reham Khashaba

**Affiliations:** 1 College of Food Science and Technology, Henan University of Technology, Zhengzhou 450001, China; saeed2014shs@yahoo.com (S.H.S.O.); reham.khashaba2012@gmail.com (R.K.); 2 National Engineering Research Center of Wheat and Corn Further Processing, Henan University of Technology, Zhengzhou 450001, China; 3 Faculty of Agriculture, New Valley University, El-Kharga 72511, Egypt

**Keywords:** sorghum, sorghum rheology, sorghum flour, sorghum composite bread, flour improvement, sorghum bread properties, sorghum flour processing

## Abstract

A Sorghum flour (SF) is a leading and prominent food source for humans in African countries. Recently extensive studies have been conducted on Sorghum bread (SB) or sorghum composite bread (SCB), covering various aspects. However, there are many technical challenges in the formation of SF and sorghum composite flour (SCF) that impact the quality of the bread and fail to meet the consumer’s desires and expectations. This review primarily focuses on the characteristics of SF, SCF, SB, and SCB, with discussions encompassing the rheological and morphological properties of the dough, improvement strategies, and bread quality. Moreover, a comprehensive analysis has been conducted to investigate the behavior of SF and SCF along with a discussion of the challenges affecting bread quality and the strategies applied for improvement. The significant demand for nutrients-rich and gluten-free bread indicates that sorghum will become one of the most vital crops worldwide. However, further comprehensive research is highly demanded and necessary for an in-depth understanding of the key features of SF and the resulting bread quality. Such understanding is vital to optimize the utilization of sorghum grain in large-scale bread production.

## 1. Introduction

Sorghum grain (*Sorghum bicolor* L. Moench) belongs to the grass family. In 2023, the global production of sorghum is projected to reach approximately 60.06 million metric tons, according to the United States department of agriculture (USDA) [1]. Sorghum is the fifth most cultivated crop globally, following wheat, corn, rice, and barley [2,3,4]. Sorghum grains and sorghum flour (SF) are rich sources of bioactive compounds, micronutrients or macronutrients, as shown in Table 1 [5]. Furthermore, sorghum is rich in proteins, minerals, vitamins, and phenolic substances [6], rendering it an anti-cancer and essential for human health. Some functional properties of SF, (1) water Absorption: SF has good water absorption capacity, which is essential for hydration and binding in food products. It can absorb and retain water, leading to improved texture and moistness in baked goods and other food formulations [7]. (2) Binding and Texture: Due to its unique protein composition, SF exhibits binding properties, contributing to the formation and stabilization of food structures [7,8]. (3) Fat Absorption: SF has the ability to absorb and retain fats, oils, and emulsifiers. This property is valuable in bakery products, as it can enhance the volume, tenderness, and shelf life of bread, cakes, and pastries [7]. Sorghum can be cooked in various forms, including porridge, alcoholic drinks, bread (gluten-free bread), and baked in Sudan in a thin form called kissra bread [9].

The integration of sorghum in the bread industry has gained remarkable attention in food industry research. Several factors contribute to the increased utilization of sorghum in the bread industry. Firstly, the gluten-free nature of sorghum enables the production of gluten-free bread [10,11]. Due to the increase in the number of people with glucose intolerance (celiac disease), SF products have increased globally, affecting 1 in 100 people worldwide. Additionally, the utilization of sorghum drastically reduces import costs for countries relying on wheat imports to meet their nutritional needs [12,13,14]. Researchers have extensively studied the rheological, morphological, pasting, and other properties of SF and sorghum composite flour (SCF). Due to the absence of a gluten network, numerous studies have focused on manufacturing and developing SF with other grains such as wheat, rice, barley, corn, and legumes. This strategy is due to the core’s solidity, the kafirin’s characteristics, and the crust’s formation [15,16]. Unfortunately, the lack of gluten in sorghum has led to a suboptimal texture of bread and other products, which requires improvement using different strategies [17]. Therefore, significant efforts have been made toward improving the functional properties of SF, including both refined and whole-grain flour.

Usually, the method employed to modify SF involves the addition of hydrocolloids, including starch, gums, protein, etc. [18]. Moreover, physical alternatives such as microwave treatment, dry heat treatment, and extrusion treatment have garnered substantial interest from researchers to enhance SF. These processes meet the manufacturing requirements for automated control, energy efficiency, and high productivity. Notably, these treatments are straightforward and inexpensive, predominantly driven by specific pressure and mechanisms that induce structural changes and interactions within the grain or flour [9,19,20,21]. Another advantage of heat treatments is their role in mitigating microbial risks for sorghum grains [22]. To enhance SF to produce sorghum bread (SB) or sorghum composite bread (SCB), it is vital to use different heat treatments of sorghum grains or SF for different industries. On the other hand, research gaps must be mentioned while preparing SB or SCB bread, such as flavor, texture, formulation, and nutrition, which must be taken into consideration. Although SF imparts the desired texture to gluten-free bread, achieving an ideal crumb structure is important. Softness remains a challenge, as does the earthy flavor [23].

**Figure 1:**
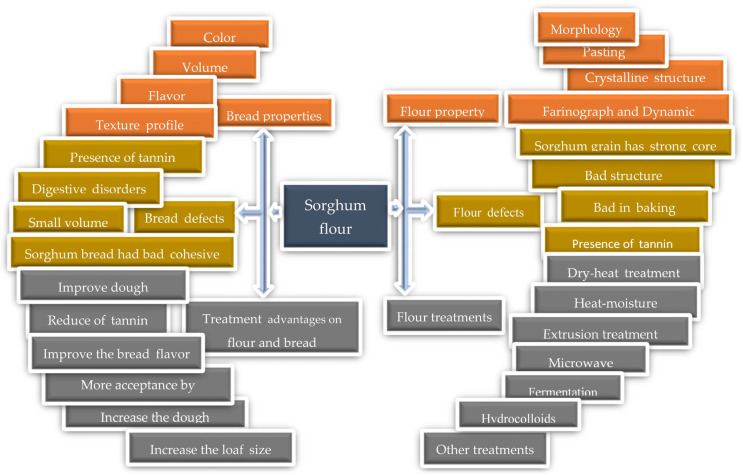
Frame graph of the reviewed content.

Few reviews have discussed the properties of SF and SFC and their role in the baking industry, including the treatments used to improve the final product. Also, certain earlier reviews discussed processing techniques for sorghum-based products in general. This includes SB as well as its relationship with glycemic and antioxidant responses [24,25]. In general, many papers studied the properties of sorghum (nutritional and rheological) after applying various treatments. This review aims to provide a systematic study on the recently scattered data, reviewing the problem of dough characteristics of SF and SCF, alongside the attribute of SB and SCB (with a focus on sorghum-wheat combined bread). This endeavor aims to propel their advancement and application in manufacturing bread and other baked goods utilizing sorghum. Figure 1 shows us the steps and path of this paper.

## 2. Rheological and Structural Properties of SF and SCF

This section explains the characteristics of SF and SCF, and the challenges encountered when forming dough. In the next section, the problems of SB and SCB were discussed in terms of color, flavor, volume, and consumer acceptance, and then passing through the section on flour improvement strategies, this section highlights the improvement of flour in turn to produce bread optimally.

Studying the technical characteristics, including the rheological properties of SF and SCF, contribute to understanding the problems of making bread to improve technical and nutritional aspects. Therefore, many researchers investigated the rheological and other techniques properties of SF and SCF dough, such as pasting, rebound, deformation, creep and creep recovery, etc. [21,26,27,28,29]. In the following section, we scout the rheology and techniques properties of SF and SCF dough and the devices used to detect it.

### 2.1. Rheological Properties

Rheological tests are among the most practical approaches for determining quality and texture indications in food items. Dough rheological characteristics define how it may deform, flow, or rupture when stressed and might be utilized in choosing and specifying suitable materials. Understanding the essential rheological characteristics of any dough can provide insight into how the dough will behave in different processing conditions.

#### 2.1.1. Dynamic Rheometry

Dynamic rheometry has been used extensively to explain the rheological characteristics of food substances, such as doughs, which provides data regarding the flow and elastic characteristics of materials. The sample for dynamic measurements is typically sandwiched between two circular plates. The system is kept at the desired temperature while applying sinusoidal stretching with various frequencies [30]. The specimen’s elastic strength relates to the storage modulus, which reflects the energy kept throughout deformation; meanwhile, the loss modulus, which reflects the energy lost throughout deformation, is connected to its viscose energy. In sorghum doughs, it has been noted that lower moisture levels cause a lack of consistency and flexibility, while greater amounts of moisture only produce a batter-like consistency. The dough’s qualities did not change after kneading, so it easily fell apart. Although adding squeezed yeast improved the rising of the dough, the loaf fell apart while baking. It was found that adding starch or gum to the dough improved its performance and produced loaves of good quality [31]. The rolling characteristics of sorghum dough were examined regarding its physiochemical characteristics, and it was also found that higher peak viscosities, fewer gelatinization temperatures, and fewer setbacks, according to Brabender Viscograph, are all indicators of good rolling quality [32]. Higher water absorption at 70 °C was linked to starch damage in the flour and had no connection to the rolling quality, which was decided subjectively. Less gelatinization temperatures result in greater gelatinization, leading to better adhesive doughs that can be easily rolled. Mixing flour and hot water in an equal proportion has been suggested. Over the years, sorghum-based SCF has been investigated in bread production to address the issues caused by a lack of gluten [33,34]

#### 2.1.2. Pasting Properties

Pasting characteristics indicate the changes that take place in the flour when applied heat while present water [35]. Pasting properties of the flours, including SF, are often measured using a Brabender Viscograph-E or Rapid Visco Analyzer (RVA) device [19,36]. Table 2 shows the farinograph and pasting properties for SF and SCF. The viscosity property is related to the amount of starch in the flour, whether in SF or SCF. In the food industry, flour’s pasting properties help select their potential applications [37]. The texture, digestibility, and end-use of a food product are influenced by its pasting properties [38]. Liu et al. [39] found that flour’s pasting properties are influenced by its nutritional composition, such as fiber, lipids, protein, and starch. Ocheme et al. [38] reported that the pasting attributes decreased as carbohydrates decreased. Protein affects the pasting properties by interacting with starch molecules and modifying their gelatinization behavior [40]. For example, in SCF, using the Brabender Viscograph-E device, the SF composite with wheat flour in different proportions sorghum: wheat 10:90, 20:80, 30:70, the maximum viscosity was high in the higher percentage of replacement of wheat flour so that the replacement sample by 30% decreased at a low level of 505.00 BU [14]. However, when replacing with proportions of 10% and 20%, no significant difference was observed in viscosity compared with 100% wheat flour [14]. The composite flour’s maximum viscosity decreased due to the sorghum starch granules’ decreased tolerance to internal pressure. The low viscosity setback during dough cooling also denotes a reduced potential to set back [41]. The matter was not much different in a similar study using the RVA instrument. There was a noticeable increase in the peak viscosity for the compound flour with a ratio of 30:70 sorghum: wheat flour [42].

As for the pasting properties of SF without other flour, an increase in the breakdown viscosity was indicated. For instance, when measuring the pasting properties of Zimbabwean SF (Macia) using the Viscograph-E device, the breakdown viscosity is very high (760.00 BU) compared with wheat flour (724.00 BU) [14]. With the same cultivar of SF, but using an RVA device, the breakdown viscosity of the SF was (426.00 cp) less than the wheat flour, which was (884.00 cp), and the peak time for SF was 6.07 min and 6.20 min for wheat flour [42]. Moreover, some heat treatments, such as microwave treatment, can increase the gelatinization temperature and thus increase the peak time of SF. We discuss this in a later section.

#### 2.1.3. Farinographic Characteristics

The Farinograph is used to measure the shear and viscosity of a dough mixture (flour and water) to provide important information about dough, such as dough viscosity, water absorption, and flour stability. The absence of gluten protein in sorghum decreases water absorption and dough development time [14]. It was observed that mixing SF with wheat flour in different proportions resulted in a noticeable decrease in water absorption in the compound dough. This decrease increases with the increasing percentage of SF due to the aforementioned reasons. In addition, sorghum bran is a good fiber source, which significantly hinders water absorption, and sorghum proteins also contain hydrophobic kaffirins [42]. The results of the Farinograph analysis of white SF mixed with wheat flour in the proportions of 10:90, 20:80, and 30:70 sorghum: wheat, showed a significant decrease in the stability, water absorption, and development time, respectively. Moreover, there are a considerable increase in the dough’s cohesion level and the dough’s resistance to deformation and a decrease in the elasticity and extensibility of the dough [43] (Table 2).

Some treatments for SF, such as extrusion, can lead to increased water absorption and dough growth for the SCF. For example, extrusion of SF at 110 °C and 160 °C with humidity of 10%, 14%, and 18% and mixing it with wheat flour in a ratio of 10:90 sorghum wheat increased water absorption and growth time of compound dough while decreasing dough stability [21]. The increase in water absorption is due to the rise in temperature and moisture of the feed. Furthermore, extrusion cooking of SF affects the stability of the compound dough because the granules are affected [26].

**Table 2:** Farinograph and pasting properties of SF and SCF.

Parameter	Flour Type	Proportions of SF	Properties	References
Pasting property	SCF	10%, 20%, and 30% with wheat flour	In the 10% replacement there is no significant difference in pasting properties like in 20% and 30% replacement rates.	[14]
	SF	SF 100%	SF has the increase in the final and breakdown viscosity, while it can decrease in peak and trough viscosity compared with 100% wheat flour.	[14,42]
	SCF	SF and Mellit flour with ratio 50:50, 75:25, and 25:75 and 100% SF and 100% millet flour as control	The highest values of peak viscosity, final viscosity in SF100%, and setback viscosity in composite flour of SF25%–Mellit 75%	[44]
	SCF	2.5%, 5%, 7.5%, and 10% of SF	Pasting parameters increase with increasing degree of substitution. The effect of tensile strength and the appearance of deformations in the dough.	[45]
	SF	SF 100% (Improved sorghum flour)	Perfect pasting properties.	[46]
Farinograph	SCF	10%, 20%, and 30% with wheat flour	Compared with the replacement proportions with 100% wheat flour, it can be found that the water absorption, dough development time, and stability decreased whenever the percentage of replacing wheat flour with SF was high; in contrast, the degree of softening it can increase with the increase in the percentage of replacement.	[14,43]
	SCF	5%, 10%, 15%, and 20% with two kinds of SF (decorticated SF or whole grain SF) combined with wheat flour	Farinograph quality decreases directly as the proportion of SF in wheat flour increases.	[47]
	SCF	30%, 40%, or 50% of red and white SF with wheat flour	Studies have shown that increasing the proportion of SF in the dough can result in reduced water absorption and stability time and increased breakdown on the Farinograph. This means that the dough becomes more difficult to mix and handle as the proportion of SF increases.	[48]

Note: SF refers to sorghum flour, SCF refers to sorghum composite flour, and SEM refers to scanning electron microscopy.

### 2.2. Structure Property

#### 2.2.1. Morphological Property

**Figure 2:**
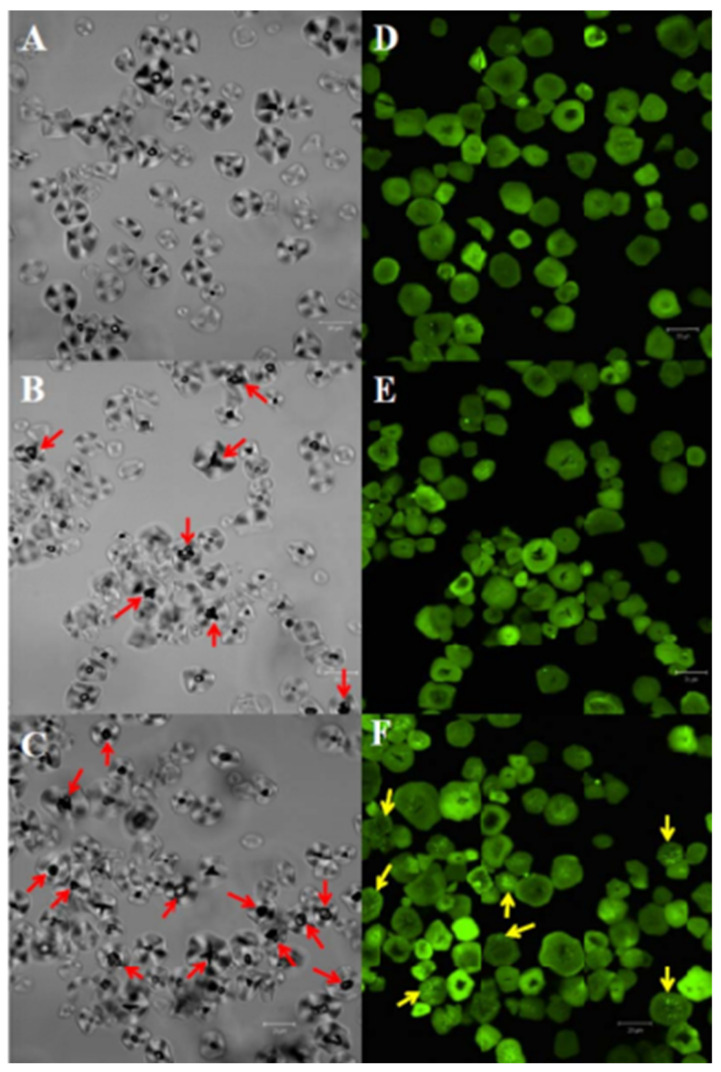
Bright-field (**A**–**C**) and CLSM (**D**–**F**) micrographs of sorghum starch granules: (**A**,**D**) starch isolated from untreated flour (no heat treatment); (**B**,**E**) heat-treated (200 g kg^−1^ moisture content, 100 °C, 4 h) starch from untreated flour; (**C**,**F**) starch isolated from heat-treated (200 g kg^−1^ moisture content, 100 °C, 4 h) flour. Red arrows indicate cracks observed in the starch hilum region and while yellow arrows point to starch-channel proteins. (Adapted from Ref. [51] with permission of Wiley. copyright 2017).

**Figure 3:**
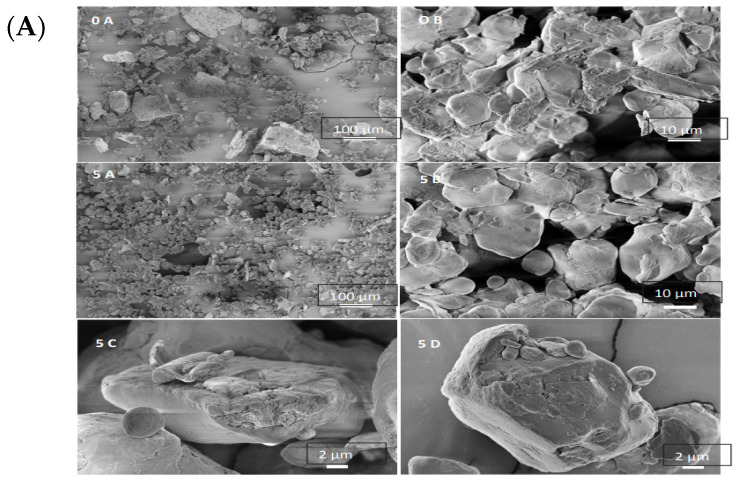

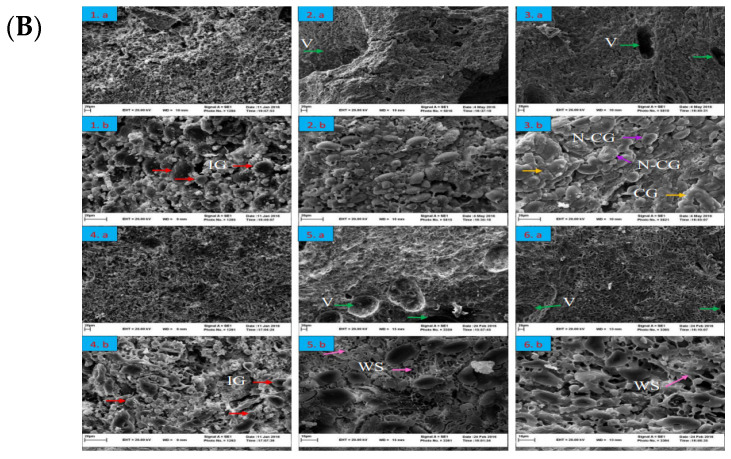
(**A**) SEM images of untreated sorghum flour (0 = no treatment) and highly milled sample (5 = 5.84 kJ/g. (Adapted from Ref. [50] with permission of Elsevier Ltd. copyright 2017)). (**B**) Scanning electron micrographs of sorghum-wheat composite dough (SWCD) at 500× (a) and 2000× (b) magnification. 1: Non-extruded SWCD; 2: Non-extruded SWCD containing 0.5% xanthan gum; 3: Non-extruded SWCD containing 1% xanthan gum; 4: Extruded SWCD; 5: Extruded SWCD dough containing 0.5% xanthan gum; 6: Extruded SWCD containing 1% xanthan gum. IG: Intact starch granules; V: Void; CG: Covered starch granules; N-CG: Non covered starch granules; WS: Web-like structure. (Adapted from Ref. [27] with permission of Elsevier Ltd. copyright 2018). (For interpretation of the references to (**B**) in this figure legend, the reader is referred to the web version of this article).

Morphological properties such as scanning electron microscopy SEM, confocal laser scanning microscopy CLSM, etc., can contribute to understanding the properties of SF and SCF dough, including particle shape, size, and surface characteristics, as well as the distribution of associated proteins with starch granules. This information can be useful in developing and optimizing food products that incorporate SF and SCF [49,50,51]. The use of CLSM can provide clear images of starch granules extracted from heat-treated SF, allowing for an examination of their morphology and any changes that may have occurred due to processing. In one study, bright-field images of the starch granules from heat-treated sorghum flour were obtained using CLSM, which showed an augmentation of the void at the hilum area in many granules in both the sorghum starch and the isolated sorghum starch samples, indicating changes in the morphology of the starch granules due to heat treatment. The void at the hilum area refers to a space or cavity that exists in the center of some starch granules. The augmentation of this void suggests that the heat treatment caused changes in the starch granules, such as water-annealing or gelatinization, which can affect the structure and properties of the starch granules [51] Figure 2 and Figure 3 shows the properties of SEM and CLSM, for SF, and SCF.

The extended vacuum observed in the starch granules of heat-treated SF in the previous statement is believed to have been created by the water-annealing of starch in a granule in conjunction with high pressure of hot water vapor at the hilum. This process can lead to changes in the morphology and properties of the starch granules, as mentioned earlier. Confocal images of the starch granules labelled with the fluorescent protein plumb-line (CBQCA) in Figure 2D–F revealed that the distribution of associated proteins with starch granules varied depending on the degree of heat treatment. Specifically, channel proteins were absent from the separated sorghum starch and the heat-treated sorghum starch, with only 1.7 and 1.9 g kg^−1^ protein being present, respectively [51].

SEM can provide a comprehensive picture of the state of starch granules and fiber in SF and SCF dough, as well as any changes that may occur due to processing. For example, SEM has been used to compare the morphology of starch granules in native flour or dough versus those in processed flour or dough that have undergone different treatments, such as extrusion or grinding with planetary balls. In one study, SEM was used to examine the effect of grinding with planetary balls on the morphology of starch granules in SF. The images showed that highly treated samples had significantly smaller starch granules and fewer large particles than the untreated sample. Additionally, the images showed that endosperm pieces, fiber particles, starch granules, and protein bodies formed a conglomerate [52]. Grinding with planetary balls and extrusion treatment can significantly affect the morphology and structure of SF and SCF dough. As mentioned earlier, SEM images have shown that grinding with planetary balls can result in broken flour particles, as well as changes in the size and distribution of starch granules, fiber particles, and protein bodies [50]. Similarly, extrusion treatment has been shown to cause changes in the morphology and structure of starch granules, including an increase in size and scattered distribution due to the filtration of amylose from the starch. Additionally, proteins can bind with starch granules, increasing their size, as seen in SEM images (Figure 3B) [21,26,27,50].

#### 2.2.2. Crystalline Structure

X-ray diffraction (XRD) is a nondestructive technique used to analyze the crystalline structure of materials, including flour. XRD works by passing X-rays through a sample and measuring the angles and intensities of the resulting diffracted X-rays. The pattern of diffracted X-rays can be used to determine the crystal structure of the material [53]. In the case of flour, XRD can be employed to analyze the crystalline structure of starch, which is the main component of flour. Starch has a unique crystalline structure characterized by XRD patterns. The XRD pattern of starch typically shows a series of peaks corresponding to the crystal lattice’s different planes [26,54].

It is common to use X-ray diffraction to characterize the properties of SF and sorghum starch, whether they are native or treated. X-ray diffraction has been applied to modified SF and sorghum starch through soaking, dry heat, heat-moisture, extrusion, or chemical treatments such as acid lamination, oxidation, and acetylation [26,51,54,55]. In one mentioned study, XRD was used to analyze the crystalline structure of sorghum starch that had undergone chemical modifications such as oxidation, acid lamination, and acetylation.

The XRD pattern revealed significant differences between the modified starch and the native starch, indicating that the modifications had altered the crystalline structure of the starch [54]. Similarly, XRD has been employed to examine the crystalline structure of SF treated with different processing methods, including moisture thermal treatment and extrusion cooking [55]. The XRD pattern of native SF exhibited a typical A-type pattern characteristic of sorghum starch. However, the crystalline structure of SF changed following treatment with moisture thermal or extrusion cooking, resulting in a pattern with A-type crystalline peaks at 2θ of 15°, 17°, 18°, and 23°, which is similar to the pattern of ordinary grain starch. The density of the A-type peaks decreased for heat-treated samples, indicating that the crystalline structure of the starch had been modified by the treatment [51]. Extrusion cooking also induces changes in the crystalline structure of SF, resulting in a pattern with V-type crystal peaks at about 2θ of 13° and 20°, indicating some starch gelatinization due to extrusion cooking [26]. Overall, XRD proves to be a valuable tool for studying the effects of different treatments on the crystalline structure of SF and sorghum starch. Through the analysis of XRD patterns, it is possible to gain more insights into the changes in the structure and properties of the starch and flour induced by different treatments.

## 3. Challenges of Native SB and SCB

SB has become a topic of interest now. It is also gluten-free because of its many benefits and contains high levels of phenolic compounds and antioxidants. On the contrary, antinutritional elements in SF, including enzyme inhibitors, tannins, phytate, or protein crosslinkers, are responsible for reducing protein digestibility [56]. Despite possessing all nutrients present in wheat flour, and even superior to it, SF is in the proportions of phenolic compounds and antioxidants. However, it is gluten-free, making SF less suitable for the bread industry because gluten plays a critical role in shaping the final bread product, giving the network structure. This network results from the interaction between glutenins and gliadins through covalent connections like disulfide bonds or non-covalent interactions like ionic compounds, hydrophobic bonds, and hydrogen bonds [57]. The role of glutenin and gliadin in the baking process is that glutenin contributes to dough resistance (dough elasticity), while gliadin provides dough extensibility and viscosity when hydrated [58]. When making bread, these properties are reflected in sufficient flexibility related to the gas retention capacity (resistance to deformation), resulting in brown bread. As for the expansion of gas bubbles during fermentation, it results from sufficient expansion of the dough [59]. Therefore, mixing SF with wheat flour to produce bread with specifications close to wheat bread is indispensable.

Sourdough bread (SDB), flatbread, kissra bread, khamir bread, and frybread are among the various bread types of sorghum [20,60,61,62]. Nevertheless, due to the lack of gluten in sorghum grain and SF structure, the dough of SF has a poor textural quality [21,63]. As a result, several academicians have developed approaches to enhance the quality of SB-based bread. As for SCB, mixing SF with different types of grains, legumes, and other starches provided the SF additional advantages and its exit from the circle of limited uses, such as using it as native bread or other industries like making local bread in Sudan (kisra) and local fermented drinks [64,65,66,67]. Among the cereals and crops used with sorghum to produce bread are wheat, cassava, potatoes, and corn [14,27,68]. SCB mixed with wheat flour is the most common for the aforementioned economic reasons [14]. The addition of Zimbabwean SF (Marcia) to wheat flour in the proportions of 90:10, 80:20, and 70:30 wheat-sorghum flours resulted in the production of a composite bread with good sensory acceptability, especially the bread produced in the ratio 90:10 did not differ much with control bread [14]. On the other hand, chickpea and cowpea integration in SCB resulted in the lowest hardness (23.91 N and 18.60 N, respectively). At the same time, the most significant protein and ash contents were (7.17 g/100 g sample and 2.72 g/100 g sample, respectively). Compared with the control bread, the legume-fortified SCB had considerably greater specific volumes and general acceptability (7.36) [68]. As a result, establishing a proper technique is critical for producing high-quality SB or SCB. Table 3 shows the characteristics of SB and SCB.

### 3.1. Color

**Figure 4:**
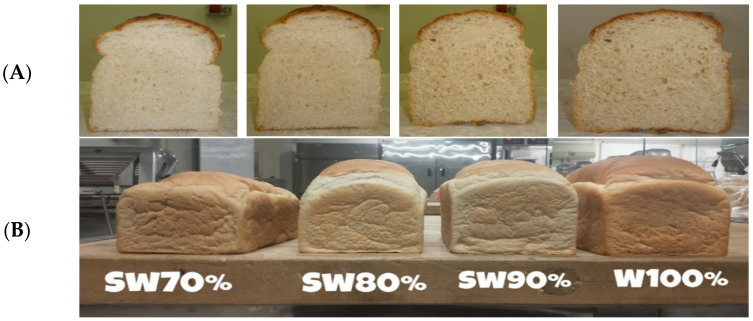
(**A**) Crumb color and pore regularity of cut product from left: 100% white standard flour; 10% white sorghum; 20% white sorghum; and 30% white sorghum [43]; (**B**) Sorghum-wheat composited bread at different proportions (Wheat 100%, Sorghum-wheat 90%, Sorghum-wheat 80% and Sorghum-wheat 70%). (Adapted from Ref. [14] with permission of Wiley copyright 2021).

Color is one of the essential factors in determining bread quality because it is one of the first characteristics that draw the consumer’s attention. The bread color measurement refers to measuring the colors of the surface and crumbs. There is no significant difference in the color of SB with different hybrids, but some flour treatments can change the color of the bread; this is what we will discuss later [20,72]. On the other hand, mixing SF with different types of flour, such as wheat flour, can produce bread with darker crumbs and crust colors. Hence, it is necessary to consider the proportion of SF added to wheat flour, 30% and 40% are considered one of the highest percentages of SF added to wheat flour [14,43,73,74]. Bread can be produced using modified SF (mosof) mixed with wheat flour. Studies have shown that adding 30% of modified SF to wheat flour can produce darker-colored bread. Modified SF is a type of sorghum that has been subjected to various pretreatments, such as soaking, germination, and/or fermentation, to improve its functional and nutritional properties [75]. The darker color of the bread produced with modified SF and wheat flour can be attributed to the presence of pigments in the SF, such as anthocyanins and tannins. These pigments can contribute to the bread’s color and also have potential health benefits, such as antioxidant activity [75]. Notably, the use of SF as a partial substitute for wheat flour produces dark-colored bread compared with wheat bread, but there are exceptions to some types of sorghum used, the quality of the bread produced, and the treatment method [69]. Figure 4 shows the crumb color and size of the bread made of SF with wheat flour.

### 3.2. Volume

The volume of regular SB is low compared with the size of wheat flour bread due to the gluten content of wheat flour, allowing it to absorb water (retaining softness and flexibility for a longer period). Good gluten can retain gases, which provide the bread the desired porosity and spongy characteristics [13,14,75]. The common bread made with SF and wheat flour is a composite bread that combines the unique nutritional and functional properties of both ingredients. However, increasing the percentage of SF added to wheat flour can decrease the percentage of protein in the bread. Sorghum grains generally contain lower protein levels than wheat flour, which can affect the overall protein content of the bread. Therefore, it is essential to carefully optimize the proportion of SF and wheat flour used in composite bread to achieve the desired balance between nutritional and sensory properties. This can help ensure that the bread is nutritious and appealing to consumers [43]. The gluten proteins are thought to be responsible for the difference in water absorption between cornmeal and wheat flour; as cornmeal consumption increases, the protein network becomes weaker, which can extend the growth period and lower the dough’s strength [76].

The highest proportions of SF mixed with wheat flour were (30% and 40%). It was observed that there was a reduction in the effects on the elasticity of the breadcrumbs, in addition to a reduction in the size of the bread [43,74,77]. The crumb porosity of bread is related to its specific volume. The specific bread volume can be influenced by several factors, such as flour type, mixing method, and baking conditions. In the case of wheat bread with 40% sorghum, studies have shown that the bread had the smallest specific volume and crumb pore diameters, suggesting that the bread had a denser crumb and was less porous than bread made with lower proportions of sorghum [77].

### 3.3. Flavor

Microbial decomposition, texture loss, and off-flavor development are all factors that contribute to the rapid deterioration of bread flavor and smell during storage. For many years, the topic of bread flavor has been ignored, but it is now emerging as a critical feature in the baking sector. It should be mentioned that the perception of bread flavor includes a wide range of characteristics like aroma, taste, warmth, appearance, and mechanical eating features, all of which contribute to customer satisfaction. Aroma is a crucial element in food acceptance, and it is mainly connected with volatile compounds that trigger olfactory receptors in the nasal canals. In contrast, taste is produced in response to active taste receptors in the presence of nonvolatile substances. The flavor of SB is related to the quality and method of treatment of SF [72,74]. The flavor of SCB often depends on the percentage of SF added, meaning the higher the SF percentage, the more flavor quality will be affected [14,74]. Abd Elmoneim et al. [73] found a significant decrease in bread flavor after adding 15% sorghum; however, these data agree with those reported by Sibanda et al. [43], who used the straight dough method for bread making, and found a significant drop in bread flavor after adding 20 percent sorghum. Sensory tests were conducted in addition to assessing the physical qualities of wheat-sorghum baking items, which showed promising results. Regarding scent, texture, and taste, loaves containing up to 30% sorghum were found to be comparable to control bread (100% wheat) [43]. Studies have shown that flatbreads containing up to 30% sorghum can be considered equally acceptable as flat breads made with 100% wheat flour. Flat breads are a type of bread typically made without yeast and are cooked on a griddle or flat surface [78]. However, it is noteworthy that these sensory tests were conducted in Zimbabwe and Egypt, where people are accustomed to the taste of sorghum.

### 3.4. Texture Profile Analysis (TPA)

An organoleptic examination of a bread’s texture complex, in terms of its mechanical features, is known as a texture profile analysis that measures chewiness, adhesiveness, springiness, resilience, cohesiveness, and firmness. The TPA hardness values for the breadcrumbs made with SF vary depending on the type of SF used. Studies have shown that this variation in hardness values is observed in hybrid sorghum bread, where white SB is harder than red SB [79]. The hardness of breadcrumbs can be influenced by several factors, such as flour type, dough formulation, processing method, and storage conditions. In the case of hybrid sorghum bread, the difference in hardness between white and red SB can be attributed to differences in their physical and chemical properties. White SB has a harder endosperm than red SB, resulting in a denser and harder crumb texture. Additionally, white SB contains a higher proportion of protein and starch than red SB, which can also affect the bread’s texture and hardness. However, it is important to note that bread’s optimal texture and hardness can vary depending on the intended use and consumer preferences [72,79]. Furthermore, using only 20% SF can achieve a hardness of 2.70 Newton’s [80], which can be considered a decent softness compared with bread produced using 61% SF [79]. TPA is also used to perform the shelf-life test of SF and SCF bread to determine the quality of bread in terms of softness, hardness, and cohesiveness. Cohesion can be considered one of the most important characteristics of bread quality because its decrease causes the bread to crumble and disintegrate while chewing [81].

## 4. Optimization Strategies to Improve SF, SCF, SB and SCB

In the previous sections of this review, we discussed excerpts from SF improvement techniques. In this part, we explore in detail these techniques that include external additives (starch, gum, and dextran) and some SF treatments such as extrusion, dry-heat, microwave, heat-moisture, and fermentation. Table 4 and Table 5 show more about these and other processors.

### 4.1. Exogenous Additives

Hydrocolloids are one of the most popular external additives to improve the properties of SF. It can be described as a group of food ingredients, primarily polysaccharides, and some proteins frequently used in various food products. The functional qualities of hydrocolloids in food are incredibly diverse, such as stabilizing, emulsifying, thickening, gelling, and regulating the formation of sugar and ice crystals. However, the two primary functions for which hydrocolloids most frequently used are thickening and gelling. Examples of primary hydrocolloids include starch, guar gum, locust bean gum, xanthan gum, carboxymethyl cellulose, and gum arabic [82].

**Table 4:** Improvement strategies applied to sorghum and their impact on the properties of SF and SCF.

Treatment Type		Flour Type	Treatment Method	Effects	References
Hydrocolloids	Starch	SF	SF with rice, potato, maize, and cassava starch, in different proportions.	Increased starch content can transform batters from soft doughs to more thin pourable batters.	[81]
		SCF	SF with native and modified cassava starch with different percentage and amylase. The following are the modified cassava starch The first mixing is 17% pregelatinized starch, 83% SF, 100% water. The second mixing is 17% native starch, 83% SF, 100% water. The third mixing is 30% original starch, 70% SF, 80% water.	Increasing the amylose concentration leads to an increase in the maximum compliance in the creep phase. Increase in the viscoelastic state and steady-state compliance in the recovery phase, but it leads to a decrease in the resistance of the mixture to deformation.	[29]
	Gums	SCF	SF, whole wheat flour, inulin, and guar gum.	Results showed that increasing the amount of sorghum flour led to increase in the dough hardness.	[83]
		SF	SF with arabic gum, guar gums mixing with Turkish beans.	Low dough pasting temperature.	[84]
Fermentation	lactic acid bacteria (LAB), dextran, yeast, etc.	SF	Lactic acid bacteria (LAB) and yeast	Increase in acid equivalent and decrease pH in sourdough.	[85]
		SCF	SF high tannin and SF low tannin fermented with baobab fruit pulp flour with levels 0%, 25%, 50%, 75%, and 100%.	Decrease in the water and fat absorption capacity, along with reducing the content of the tannin and ph.	[86]
		Sorghum grain	Treated grain sorghum by three methods: Fermented grain sorghum by LAB. Fermented and steaming of grain sorghum. Fermention, flaking and steaming grain sorghum	Fermentation process can increase protein content. Fermented and steaming SF can reducing the content of tannin. Fermentation, flaking and steaming of grain sorghum can increase the anti-oxidants.	[87]
		SCF	70% SF with 30% potato starch and hydroxypropyl methylcellulose	The sourdough has a high resistance for the deformation than non-sourdough dough.	[88]
Heat-treatments	Microwave treatment	Sorghum grain	Microwaved SF at 350 and 500 watts for 15, 30, and 45 Seconds	Increased phenolic and antioxidant content.	[22]
		Whole sorghum kernels	Microwaved sorghum grain at 36 and 90 kJ/100 g.	The high stability of the flour when stored even at high temperatures due to a decrease in the level of fat.	[89]
		SF from sorghum grain treated with microwave	Microwaved at 600 W for 6 min.	It can increase the proportions of dietary fiber contents (soluble and insoluble). Decreasing in pasting viscosity with increase in its temperature and time. Decreasing in the level of fast-digesting starch, while an increase in the content of slow-digesting starch.	[19]
	Heat-moisture treatment	SF with sorghum starch	Heat-moisture treatment at 25% and 20%.	Structure of phenotypic of starch gel much organize, small size starch granules High crystallization rate, lower adhesive viscosity Significant effect on swelling strength and rebound viscosity.	[90]
		SF	Heat-moisture treatment at moisture contents (0, 125, 200, and 300 g kg^−1^ w.b), temperatures (100, 120, and 140 °C) and times (1, 2, and 4 h)	Increased the content of resistant starch.	[51]
		Sorghum grain	Heat-moisture treatment at moisture content at 17% with 100 °C for 4 h.	Increased oil and water absorption capacity. Increased activity of antioxidants.	[91]
		Sorghum starch	Sorghum starch treated with moisture content at 18% to 27%.	After heat-moisture treatment, of the starches’ ability to absorb water, oil, and alkaline water can be improved.	[92]
	Extrusion cooking treatment	SCF	Extrude SF at 110 °C and 160 °C die temperature and moisture at 10%, 14% and 18% and mixing with wheat flour.	Decreased dough stability while increasing water absorption and dough development time. Increased dough stiffness compared with dough containing non-extruded SF. Increased the crumb heating rate. Extruded sorghum dough shows starch granules less stable compared with the original dough	[21]
		SF	Extrude at 110 °C and 160 °C die temperature and moisture at 10%, 14% and 18%	Increasing starch crystallization, and maximum gelatinization temperature (TP). SEM shows starch granule shape of extruded SF are larger than original SF	[26]
		SF	Extrude at 110 °C and 160 °C die temperature and moisture at 10%, 14% and 18%	Reduced the sandy properties of sorghum	[93]
		SF	Extruded SF and soybean flour	Decreased tannin content. Increase the content of fat, calcium, iron, fiber, crude protein, and moisture	[94]
		Sorghum grain with barley	Sorghum grain and barley with different particle size and extrude at 100 °C and 140 °C.	The pressure which generates through the extrusion process can be higher for soft fraction of sorghum however lower for the barley. When using extrusion at the lower temperature can result in a higher final paste coherence and lower water absorption indicator.	[95]
Others treatments	Malted sorghum grain	Sorghum grain	Treating the malt by using four methods drying the malt at high temperatures, steaming, stewing and boiling before the drying, and then add to the SF.	Malting can raise the water-holding capacity of SF. Malting can lower the pasting temperature of SF to be near of the wheat flour value; however, the paste viscosity it can be lower.	[96]
	popping process	Sorghum grain	SF from sorghum grain treated with popping process	Partial or complete gelatinization of the starch occurs after the popping process. There is an increase in the thermic stability of popped sorghum according to the viscosity profile.	[19,36]
	milling process	Sorghum grain	SF from sorghum grain modified by Planetary ball milling	It can lead to a positive change in the functional properties of SF. It can increase the content of damaged starch. SEM shows the change that occurs in starch granules and broken flour particles as a result of the ball milling process, so it can see protein and fiber bodies clearly	[50]
	Nixtamalization and cooking	Sorghum grain	Nixtamalization (alkaline cooking) and cooking	Nixtamalization one of the most efficient process for lowering of tannins. While the gallic acid it can consider to be one of the important bioaccessible phenolic compounds.	[97]
	Treatment by ultrasonication	SF from whole sorghum grain	Using ultrasonication for treatment sorghum for 10 min at 40% capacity.	Using ultrasonication to treat SF can enhance the digestibility or biological characteristics.	[98]
	High pressure treatment	SF	SF dough modified at pressures of 200 to 600 Megapascal [Mpa] at 20 °C	Cause of pressure-induced starch gelatinization, bunt consistency may rise at pressures of 300 Megapascal [Mpa].	[99]
	Boiling	Grain sorghum	Boiling	Enhance the biological value of sorghum grain.	[87]
	Different treatment	Grain sorghum	Different treatment (dry heat, bursting, control, grind, wet cooking with and without water and wet cooking in pressure) at the same time.	The anthocyanins, phenols, and the content of protein can all be preserved by dry heat, which also exhibits between 94% and 95% of the radical scavenging activity. Using heat-treatment and merging with pressure, can enhance natural hydrolysis of proteins.	[100]
	phosphorylation	SF	phosphorylation of SF	A decrease in the viscosity of the modified SF, an increase in the breakdown viscosity of the modified SF and low setback of the modified SF compared with the original. The swelling power and water solubility of phosphorylated SF increased with water temperature.	[101]

Note: SF refers to sorghum flour, SCF refers to sorghum composite flour.

**Table 5:** Improvement strategies applied to SF/SCF and their impact on the properties of SB and SCB.

Treatment Type		Method	Principle of the Treatment	Properties of Product	References
Hydrocolloids	Starch	SF	SF with 10% rice starch from different sources as a flour	Loaves with better crumb grain.	[18]
		SF	SF with different starches and xanthan gum	The bread had better texture/mouthfeel according to sensory analysis. Xanthan gum can enhance the dietary fiber content of chemically sourdough gluten-free sorghum bread.	[102]
		SF	SF with starch and xanthan gum	better texture/mouthfeel for bread according to sensory analysis	[103]
		SF	SF with rice, potato, maize, and cassava starch, in different proportions.	Increasing the starch content of all baking’s can reduce crumb chewiness and firmness while increasing resilience, springiness, and cohesiveness.	[81]
		SCF	SF with native and modified cassava starch with different percentage and amylase. The following are modified: cassava, starch 1. The first mixing is 17% pregelatinized starch, 83% SF, 100% water 2. The second mixing is 17% native starch, 83% SF, 100% water 3. The third mixing is 30% original starch, 70% SF, 80% water	Also, the breads with native starch can have better crumb characteristics compared with the breads containing pregelatinized starch. Increasing the condensation of enzymes can lead to reduce the crumb firmness, chewiness, springiness, resilience, and cohesiveness, while rising the adhesiveness.	[29]
		SF	The whole grain red SF was incorporated into the gluten-free SB formula by substituting corn and potato starches (10%, 20%, 30%, and 40%; *w*/*w*).	In comparison with the control, SB 30% and SB 40% had improved technological parameters, including higher specific volume and softer and better-colored crumb. Additionally, SB that had a higher whole grain SF proportion was more well-liked by consumers.	[104]
	gums	SF	SF with xanthan gum, rice, potato, tapioca starch and hydroxypropyl methyl cellulose.	Good texture and mouthfeel for bread.	[102]
Fermentation	Lactic acid bacteria (LAB) and yeast, etc.	SF	Lactic acid bacteria (LAB) and yeast	The proximate structure of the SB samples can show more increase in the moisture, ash, protein, moisture, and fat content.	[85]
		SCF	SF sourdough made with LAB and different levels of dry nabag pulp powder (1%, 3%, 5%, and 7%).	It led to the production of bread with excellent specifications, according to sensory evaluation	[105]
		SCF	Fermentation of lima bean after traditional steeping and mixing with SF and wheat flour	The bread produced with treated lima bean have good sensory properties, according to sensory evaluation.	[106]
Heat-treatments	Dry-heat treatment	SF	Dry-heat SF by oven at 125 °C and 90 °C for 15, 30, or 45 min.	Good acceptance for bread according to the consumer test. Larger size for bread compared with native bread.	[20]
	Extrusion cooking treatment	SF	Extrude at 110 °C and 160 °C die temperature and moisture at 10%, 14%, and 18%	A high volume of the SCB and more density.	[93]
Other treatments	Malted sorghum grain	Sorghum grain	Treating the malt by using four methods drying the malt at high temperatures, steaming, stewing, and boiling before the drying, and then add to the SF.	The crumb of the bread making from (30%) boiled malt flour can have good characteristics than other bread.	[96]
	High pressure treatment	SF	SF dough modified at pressures of 200 to 600 Megapascal [MPa] at 20 °C.	The quality of the SB which have various amounts of modified SF at 200 Megapascal [MPa] was not significantly different compared with unmodified bread.	[99]

Note: SF refers to sorghum flour, SCF refers to sorghum composite flour, SB refers to sorghum bread, and SCB refers to sorghum composite bread.

#### 4.1.1. Gums

**Figure 5:**
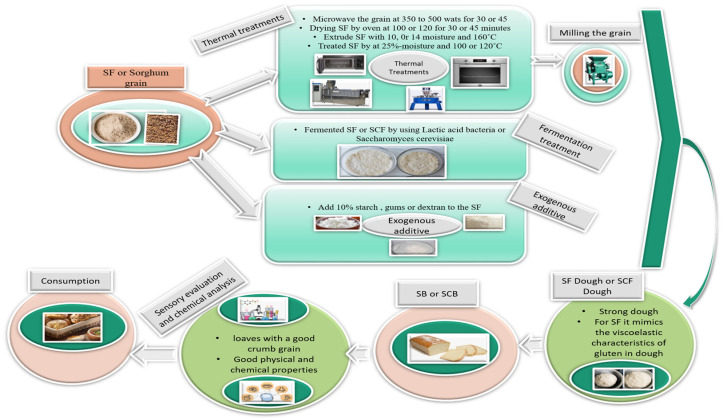
Graphic diagram of various processes for improving SF and producing SB and SCB.

Recently, several studies focused on using colloids in sorghum instead of gluten to improve its rheological properties. Xanthan gum is one of the most popular colloids used in dough improvement, is a polysaccharide that can form a gel-like structure in water, which can help improve the dough’s structure and texture. In one study, xanthan gum was added at 0.5% and 1% on SF combined with wheat flour in a 90% wheat:10% sorghum ratio. The results showed that adding xanthan gum positively affected the properties of composite flour and bread, producing a stronger dough [27]. However, in another study, sorghum was extruded at 10% feed moisture and 160 °C and mixed with wheat flour, and xanthan gum was added in the same proportion (0.5% and 1%). The results showed that adding xanthan gum led to a reduction in the strength of the dough and an increase in the hardness of breadcrumbs. The difference in the effect of xanthan gum on the dough properties can be attributed to the differences in the processing method and the proportion of SF used. The extrusion process can affect the properties of SF, which can influence the behavior of xanthan gum in the dough. Additionally, the proportion of SF used in the composite flour can affect the dough’s rheological properties and the overall quality of the bread [27]. To enhance this, when looking at the properties of Farinograph in this regard, it shows that the use of SF extruded increases the time of dough growth; this is consequent to the high water absorption capacity of the extruded SF [26]. However, adding xanthan gum to the non-extruded SF led to increased dough stability, increased dough growth time, and a significant increase in water absorption [107]. The resulting increase in the strength of the dough is that the xanthan gum increases the speed of the binding of starch particles and thus enhances the strength of the dough [108]. Moreover, adding xanthan gum with some different starches to fermented SF resulted in bread production with a distinctive flavor, better crumb texture, and a significant increase in fiber content [102]. In addition to xanthan gum, other natural gums such as gum arabic extracted from sources like flax seeds, okra, fenugreek, and cress seeds have also been studied for their positive effect on composite SF. Studies have shown that adding gum Arabic and other natural gums to SF can improve the properties of gluten-free dough. For example, the properties of composite dough produced by substituting 5% of gum Arabic into SF combined with Turkish bean powder increased the fiber, ash, and moisture content in gluten-free dough [84]. Studies have shown that adding gum arabic and guar gum to low-tannin SF can improve its ability to bind oil, water, and protein solubility, improving the final product’s texture and sensory properties. Additionally, adding these gums can improve the properties of the flour according to the Farinograph test. Generally, the protein in sorghum has a lower solubility than wheat protein, which can affect its functionality in dough making and other food applications. Regarding the ability to bind water and oil, studies have shown that the addition of gum arabic with or without guar gum to sorghum dough can reduce the ability to bind oil and water, while the addition of guar gum alone can increase the binding strength. This can affect the flour’s water absorption process, as it decreases when adding gum arabic with or without guar gum and increases with the addition of guar gum alone [109]. With the gum side, some baking materials, such as baking powder, can also be used to add flavor to the product [110]. Figure 5 shows the different stages of developing SF and producing SB and SCB.

#### 4.1.2. Starch from Other Sources

Starch is a carbohydrate consisting of glucose units linked together by glycosidic bonds. The starch powder mainly comprises branched amylopectin and helical and linear amylose. Amylopectin and amylose constitute about 98–99% of the dry weight of the starch. Starch is available in food grains (rice, yellow corn, sorghum, wheat, and cassava), one of the cereals’ most significant nutritional components [111,112,113]. Producing high-quality gluten-free bread is what researchers and those interested in bakeries are looking for to meet consumer demands. Using starch and resistant starch in the production of gluten-free bread improves the rheological and organoleptic properties and can increase the nutritional properties [112,114]. Recently, many studies focused on treating starch before using it because most naturally show a lower thickening ability, weak shear resistance, and reduced swelling [115,116].

Adding starch to SF as a flour blend can significantly impact bread quality, particularly its size or volume. Studies have shown that adding rice starch to sorghum flour at a proportion of 10% as flour, along with other colloids such as tapioca starch with 3% HPMC (Hydroxypropyl methylcellulose), potato starch with 4% xanthan, and rice starch with 3% xanthan, can increase the size of the loaves compared with loaves made with 100% cornmeal. When the proportion of rice starch was increased to 20% and 30%, no further change in loaf size was observed [117]. The viscosity of the dough resulting from the addition of starch to SF can also impact the quality of the bread. Dough with lower viscosity tended to have better crumb granules, while higher viscosity dough can result in coarser crumbs [117]. Furthermore, adding a mixture of nana potato starch and rice starch, enhanced with different hydrocolloids such as xanthan gum and hydroxypropyl methylcellulose (HPMC), can improve the flavor and texture of SB [18]. Increasing the amount of added starch in sorghum dough can significantly impact the texture and quality of the resulting bread. Generally, increasing the amount of starch can improve the bread’s elasticity and softness while reducing the crumbs’ hardness [81]. In conclusion, it can be said that careful optimization of the proportion of added starch and other ingredients is necessary to achieve the desired texture and quality of gluten-free bread made with SF.

#### 4.1.3. Dextran

Dextran can be defined as a bacterial exopolysaccharide (EPS) or a complex glucan in the form of chains of varying length linked together by α-1,6 glycosidic linkages between glucose monomers, from which α-1,3 linkages are branched [118]. Lactic acid bacteria LAB can produce dextran in fermented dough for bread making, called in-situ dextran production. The purpose of producing dextran vis in situ is to isolate dextran from bacteria and use it in the dough to act as a hydrocolloid by binding water and mimicking the viscoelastic properties of gluten in the dough [119,120]. The influence of dextran in the dough can depend on the type of strains used in the enrichment and the characteristics of the flour. For example, one study observed a considerable decrease in the strength of sorghum-buckwheat composite dough fermented with dextran [121]. The effect of dextran on the dough can vary based on factors such as the type of bacteria used to produce the dextran, the amount of dextran added, and the characteristics of the flour. A study was conducted on sorghum-wheat composite dough enriched with dextran produced by *Weissella cibaria* MG1, it was observed that adding 10–20% of the enriched dough can lead to an increase in the viscosity of the dough and a lower complex modulus G*, indicating a softer and more elastic texture in the resulting bread [122].

**Figure 6:**
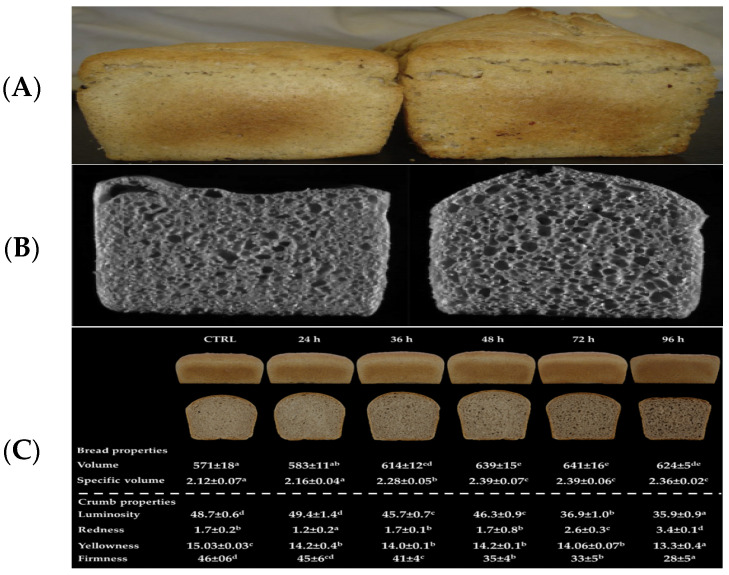
(**A**) Comparison of specific volume of bread produced from untreated and heat-treated sorghum flour. (**left**): untreated control flour; (**right**): heated treated flour at 125 °C for 30 min; (**B**) C-cell images of crumb structure for bread produced from unheated sorghum flour (**left**) and heat-treated sorghum flour at 125 C for 30 min (**right**). (Adapted from Ref. [20] with permission of Elsevier Ltd. copyright 2016); (**C**) properties of bread from wheat containing 20% unsprouted (CTRL) or sprouted sorghum at different times(24 h, 36 h, 48 h, 72 h, and 96 h). Different letters in the same row indicate a significant difference among samples (one-way ANOVA; Tukey HSD test; *p* ≤ 0.05). Volume and specific volume are expressed in mL and mL/g, respectively. Crumb firmness is expressed in N. (Adapted from Ref. [123] with permission of MDPI. copyright 2021).

Moreover, the magnificent effect of dextran produced in situ by *Weissella confusa* A16 on modifying the flavor and texture of sorghum-wheat composite dough was observed. Compared with the original bread, sorghum-wheat composite dough enriched with dextran produced in situ can produce soft, high moisture, and foldable bread [123]. This was observed in a study that used a sorghum-wheat composite dough ratio of 1:1 and enriched it with a dextran rate of 0.56% of the bread weight [124]. Figure 6 shows the comparison of specific volume of bread produced from untreated and heat-treated sorghum flour.

### 4.2. Modification of SF

#### 4.2.1. Microwave

A microwave is a device that uses electromagnetic radiation in a specific frequency range to heat materials. It is considered one of the most efficient heating devices because it can heat materials quickly and efficiently [125]. The mechanism of microwave heating is based on dielectric heating. When materials, such as food, are exposed to microwaves, the polar molecules in the material, such as water molecules, are stimulated to rotate rapidly. This rapid rotation produces thermal energy, which in turn heats the material [126]. Recently, researchers have been studying the use of microwaves, either alone or in combination with other treatment processes, such as thermal moisture treatment, to improve the properties of different types of gluten-free flour, such as rice, sorghum, and maize flour [127,128,129]. Microwaving sorghum grain can improve the quality of SF, including its color, texture, and sensory properties. For example, a study found that microwave treatment of sorghum flour at 36 and 90 kJ/100 g improved the flour’s color and texture [89]. In addition to improving the quality of SF, microwave treatment can also increase its shelf life, even when stored under high temperatures. This is because microwave treatment can disrupt the activity of flour lipase. By slowing down the oxidation of free fatty acids, microwave treatment can help extend the shelf life of the flour [89].

On the other hand, roasting sorghum grain by microwave can decrease the breakdown viscosity, peak viscosity, pasting viscosity, and setback. A study found that roasting sorghum grain by microwave at 778 to 546 kg/m^3^ led to a decrease in the pasting properties of SF, including a decrease in breakdown viscosity, peak viscosity, pasting viscosity, and setback. This may be due to the effect of microwave roasting on the starch granules in the grain, which can result in changes in the structure and properties of the starch [130]. Moreover, when three sorghum cultivars were treated with microwave and viscosity properties were measured, there was a noticeable variation in the setback values of the three sorghum cultivars [19]. This discrepancy is attributed to the polymerization temperature (DP) change due to microwaves [19,131].

#### 4.2.2. Dry Heat Treatment

Regarding dry heat treatment, it is considered scarcely used in flour processing, and the oven is often used to conduct it. Dry heat treatment of sorghum grain can be used along with particle size distribution. Some functional properties, such as water absorption capacity and increasing fat and fiber, were found to be related to the approximate composition of the flour, whereas moisture, protein, solubility index, ash, water holding capacity, and foam were not. On the other hand, heating SF at 125 °C for 30 min increased the volume of bread produced from heat-modified flour compared with control bread, and the consumer acceptance score was 5.05 for heat-treated bread and 4.76 for native control bread [20]. This can be seen in Figure 6, which shows how native and heat-treated SF bread looks and compares with SCF bread. Although several researchers have compared dry heat and heat-moisture treatments in their effect on SF properties [132], some researchers have researched the use of dry heat treatment along with some other techniques, such as the milling process (particle size distribution) that have a role in the functional properties [133,134]. Furthermore, the dry heat and pressure proved effective [100,100].

#### 4.2.3. Extrusion Treatment

Extrusion is a popular method for processing and producing various food products, including those based on grain products like cakes, pasta, noodles, and bread. Extrusion involves subjecting the flour to high temperatures and pressure, which can cause chemical and physical changes in the flour. These changes can improve the resulting food products’ texture, flavor, and nutritional quality [3,135,136,137]. It is worth noting that high-shear extrusion is a processing method that can be used to produce various food products, including flours with improved technical specifications. In the case of SF, combining it with other ingredients like oat flour, lentils, chickpeas, natto beans, quinoa, and amaranth through high-shear extrusion can produce flour with excellent technical specifications. The resulting extruded compound flour had acceptable properties and cold crystallization ability, suggesting that it can be a useful ingredient for various food products. The use of rice flour for comparison purposes is also important, as it allows for a better understanding of the unique properties of the extruded SF [138]. Moreover, extruded SF with some legumes such as defatted soybean flour with a moisture content of (15%, 18%, and 21%) and temperature (135 °C, 150 °C, and 165 °C) can lead to an increase the fat protein, iron, calcium, and moisture content while decreasing the carbohydrate, phytate acid, and tannin content [94]. The cold extrusion of soybean flour, sorghum, and wheat flour can lead to the formation of a mixture with qualities similar to peanuts [139]. Moreover, when studying the thermal properties, chemical composition, crystallization, and morphology of extruded SF at a die temperature of 110 °C and 160 °C and moisture of 10%, 14%, and 18%, there was a decrease in gelatinization temperature ranges (Tc–T0), with an increase in starch crystallization and maximum gelatinization temperature (Tp). This indicates an increase in the moisture content of the feed [26]. In the case of adding extruded SF to wheat flour, the compound dough’s elasticity decreases. The dough crumbs’ heating rate increases during baking [21], and the bread resulting from this process acquires good sensory qualities such as flavor, in addition to an increase in the volume of the produced compound bread (number of cells/cm^2^) and the moisture of the crumbs, as well as an increase in the redness of the bread. Moreover, the peel color and crumbs change to more red and yellow (a*) and (b*), respectively, while the (L*) would be light. This might be attributed to occur of the Maillard reaction, which results from the interaction between amino acids and free sugar (Table 4) [93]. Pasting properties can be affected by extrusion treatments. For example, the extrusion of SF at 110 °C and 160 °C with humidity of 10%, 14%, and 18% and mixing it with wheat flour in a ratio of 10:90 sorghum wheat, respectively, increased water absorption and growth time of compound dough, while decreasing dough stability [21]. The increase in water absorption is due to the feed’s rise in temperature and moisture. Furthermore, extrusion cooking of SF affects the stability of the compound dough because the granules are affected. Additionally, the effect of extrusion cooking on the stability of the compound dough may be due to changes in the granule structure and properties of the extruded sorghum flour. These changes can affect the behavior of the flour in various food applications and may have implications for the quality and texture of the resulting food products [21].

#### 4.2.4. Heat Moisture Treatment

The heat-moisture processing can change the physical and chemical properties of starch by inactivating the starch crystals. Thus, it leads to an increase in the crystallization of starch, as well as its effect on solubility and rebound viscosity [90]. For example, the heat-moisture treatment of SF and starch at 20% and 25% moisture content increased crystallization with a decrease in the pasting viscosities and appearance of a starch gel structure in a regular shape and small particles [90]. The decrease in peak viscosity and setback observed in the annealed starch suggests that the process can lead to changes in the properties of the starch granules, including their ability to absorb water and form a gel under specific conditions. The decrease in swelling strength also suggests that the annealing process can affect the ability of the starch granules to swell and absorb water [140]. The heat-moisture-treated starch showed a significant decrease in crystallization and amylose molecule chains. This indicates the ability of amylose in sorghum to degrade under heat and moisture stress [140]. This may reduce protein digestibility ability because of the increase in resistant starch and amylose complexes [51]. On the other hand, a noticeable increase in the antioxidant activity of sorghum treated with a moisture content of 31.72% and 51.07% at 100 °C for 4 h was observed. The oil and water absorption capacity was much higher after heat-moisture treatment [91]. Since heat-moisture treatment affects the functional properties of SF flour, high temperature can lead to discoloration of SF (decreased flour whiteness) [26].Therefore, it is imperative to control the temperature when performing heat-moisture treatment to avoid discoloration of flour and high gelatinization temperature. However, heat-moisture treatment of sorghum grain/flour significantly increases nutritional components, such as enhancing antioxidant activity and increasing the amount of resistant starch.

### 4.3. Fermentation

Various types of fermentation can be used to enhance SF or to produce bread and gluten-free bread. Lactic acid bacteria (LAB) and baker’s yeast are among the most common types of fermentation used in the production of SB and SCB or to enhance SF and SCF [85,105,124,141,142].

It is possible that the use of dried yeast with *L. reuteri* and *W. cibaria* in the fermentation of SF, along with the addition of 10% or 20% of the fermented dough, can lead to the formation of exopolysaccharides (EPS) during fermentation. EPS are complex carbohydrates that are produced by certain microorganisms during fermentation and can have a significant impact on the texture and quality of the final product. In this case, the formation of EPS during fermentation may contribute to a decrease in the elasticity and strength of the dough. This is because EPS can interfere with the development of gluten in the dough, which is responsible for its elasticity and strength. Additionally, EPS can also increase the water-holding capacity of the dough, which can further contribute to a decrease in its overall strength [122]. It was observed that the increase in the release of fructose, glucose, and sucrose helps to increase the production of yeast CO_2_. Thus, it leads to the production of bread with softer crumbs and increases the shelf life of bread [122]. The fermentation technique can be used by applying in situ-produced dextran or ex situ dextran through the fermented dough technique, as it leads to the production of bread with high sensory acceptance and a high increase in the specific bread volume [141]. Concerning the use of on-site dextran, mostly LAB strains from the genus *Weysella* that exhibit mild acidification are utilized [141]. It has been reported that sourdough fermentation of whole SF with wheat flour at a ratio of (50%:50%) using *Weissella confusa* A16 strain (dextran produced in situ) led to a significant increase in the pliable, moist, firm, soft, elastic, and smooth compared with sorghum dough or native SCB [124]. Moreover, using *Weissella cibaria* MG1 to ferment buckwheat, sorghum, teff, and quinoa flours can produce dough and gluten-free bread with high sensory quality. When compound sourdough and bread were compared with native wheat dough and bread (control), the sourdough-containing bread had higher sensory quality [121].

Germination of sorghum grains before milling, followed by fermentation by LAB, can increase the proportion of nutrients and improve the digestion of protein and starch. This process can also significantly reduce the proportion of anti-nutrients in SF, which can interfere with the absorption of nutrients [143]. Furthermore, fermentation can be enhanced by adding fruit products such as peels and pulp. For example, adding buckthorn pulp powder in different proportions to fermented sorghum dough and fermenting it at different levels increased acidity and decreased the pH. Research has shown that adding buckthorn pulp powder in proportions of 1%, 3%, 5%, and 7% to fermented sorghum dough and fermenting it at levels of 10%, 20%, and 30% increased acidity from 2.8 to 15 mL and decreased the pH from 6.30 to 3.54. However, acidity decreased when buckthorn pulp and SF were added to the fermented dough [105].

## 5. Conclusions

In general, the technical characteristics of SFs do not meet the manufacturing requirements well based on factors such as rheology, pasting, X-ray differentiation, and morphology characteristics. However, modifications and additions to SFs can alter their technical characteristics and functions, making them excellent for use in the bakery and other industries. SB and SCB have become of interest to researchers due to their ability to meet the requirements of celiac disease. Moreover, they allow for optimal utilization of sorghum, which is one of the most important cereal crops for many African countries. Sorghum is characterized by its high content of natural antioxidants, minerals, and dietary fiber; however, it also contains anti-nutritional factors. Upon reviewing the literature, it was concluded that producing sorghum bread with satisfactory quality is challenging due to the nature of SF. Therefore, various additions or improvements to flour have been explored, such as adding hydrocolloids, merging with legume powders or some and other grains flour, or treating the flour through fermentation, thermal moisture treatment, extrusion cooking, dry heat treatment, and microwave processing. Adding hydrocolloids to SF alters the dough’s rheology, affecting the properties of bread and other products. The mechanism behind hydrocolloid action involves modifying the dough’s viscoelastic behavior and extensional properties, resulting in improved bread texture quality and extended shelf life. As for the heat-moisture treatment, extrusion and dry heat have been proven to increase fiber content, particularly resistant starch, which is vital for nutrition. Moreover, fermentation improves the flavor and texture of the products. In summary, it can be concluded that utilizing different treatment techniques for SF is essential for achieving technical, functional, and nutritional purposes objectives. In conclusion, in addition to bread, it can be recommended to use processed and unprocessed SF to enhance cakes, biscuits, and school breakfast meals to add nutritional value to them.

## Figures and Tables

**Table 1:** Chemical composition of sorghum flour and sorghum grains (whole grain) (Redrawn from Ref. [5] with permission of USDA copyright 2018).

Component		Sorghum Grains (100 g)	Sorghum Flour (100 g)
Macronutrients	Water (g)	12.4	10.26
	Energy (kcal)	329	359
	Protein (g)	10.62	8.43
	Total lipid (fat) (g)	3.46	3.34
	Ash (g)	1.43	1.32
	Carbohydrate (g)	72.09	76.64
	Fiber, total dietary (g)	6.7	6.6
	Sugars, total including NLEA (g)	2.53	1.94
	Starch (g)	60	68
Minerals	Calcium (mg)	13	12
	Iron (mg)	3.36	3.14
	Magnesium (mg)	165	123
	Phosphorus (mg)	289	278
	Potassium (mg)	363	324
	Sodium (mg)	2	3
	Zinc (mg)	1.67	1.23
	Copper (mg)	0.284	0.253
	Manganese (mg)	1.605	1.258
	Selenium (μg)	12.2	12.2
Vitamins	Total ascorbic acid (C) (mg)	0	0.8
	Thiamin (mg)	0.332	0.329
	Riboflavin (mg)	0.096	0.061
	Niacin (mg)	3.688	4.496
	Pantothenic acid (mg)	0.367	0.539
	Vitamin B-6 (mg)	0.443	0.325
	Folate, total (μg)	20	25
	α-tocopherol (E) (mg)	0.5	0.5
	Phylloquinone (K) (μg)	0	6.4
Fatty acids	Total saturated (g)	0.61	0.528
	Total monounsaturated (g)	1.131	0.943
	Total polyunsaturated (g)	1.558	1.403
Amino acids	Tryptophan (g)	0.124	0.106
	Threonine (g)	0.346	0.312
	Isoleucine (g)	0.433	0.309
	Leucine (g)	1.491	1.085
	Lysine (g)	0.229	0.174
	Methionine (g)	0.169	0.145
	Cystine (g)	0.127	0.165
	Phenylalanine (g)	0.546	0.441
	Tyrosine (g)	0.321	0.225
	Valine (g)	0.561	0.387
	Arginine (g)	0.355	0.33
	Histidine (g)	0.246	0.167
	Alanine (g)	1.033	0.758
	Aspartic acid (g)	0.743	0.556
	Glutamic acid (g)	2.439	1.741
	Glycine (g)	0.346	0.313
	Proline (g)	0.852	0.651
	Serine (g)	0.462	0.411

**Table 3:** The characteristics of color, volume, and flavor of SB and SCB.

Flour Type	Proportion and Characteristics	Color	Volume	Flavor	Texture	References
SCF	Wheat flour with red and white SF with proportion of 30%, 40%, and 50%.	The addition of white and red SF to wheat flour for produce bread reduced the color value of the bread compared with 100% wheat bread. Increasing the darkening of the color of the composite bread, due to the SF containing a high percentage of phenols.	Increasing the SF ratio leads to a significant decrease in the volume of the composite bread.		The addition of white SF increases the strength of the textural. The addition of red SF provides a texture strength like control bread (100% wheat).	[69]
SCF	White flour—maida and SF with proportion 5%, 10%, 15%, 20%, and 25%.	Compared with the other ratios, wheat 100% had the best mark of 8.80 and was on par with 5% (8.20). As for the other percentages, they were different.		The increased amount of SF in blended bread reduces flavor from 8.60 wheat 100% to 3.80 SF 25%.	The degree of husk texture decreases with increasing substitution of SF in white flour	[70]
SCF	10% to 20% and 30% SF with wheat flour	Breadcrumbs containing high ratio of SF become darker or brown to gray, with a visible particles of sorghum bran.	Reduced the volume of compound bread as the proportion of SF replacement increases.	A significant difference in the flavor of the composite bread and the control bread according to the tasters’ evaluation.	The bread texture was rated as satisfactory to good according to members of the sensory analysis team.	[43]
SCF	30%, 40%, and 50% red and white SF with wheat flour	The red SF flat breads seemed darker compared with the control, which may have been caused by the red SF’s higher concentration of colored polyphenolics like anthocyanins.		The larger particle size of the SF may improve the sensory acceptability of the breads		[71]
SCF	10%, 20%, and 30% Zimbabwean marcia SF with high gluten wheat flour	Bread with 10% SF, had better color than other types of bread	Despite the use of a flour with a high wheat gluten concentration, the gluten network degraded as the amount of sorghum was increased, resulting in a reduction in bread volume.	There was no discernible difference in overall acceptability between the wheat bread and the bread with 10% and 20% SF added. The difference was in the bread, which had a 30% SF.	The bread with 10% SF added had a better texture and was chewier and more elastic than the other bread additions.	[14]

Note: SB refers to sorghum bread, SCB refers to sorghum composite bread.

## Data Availability

Data are contained within the article.
